# Dynamic Balance of Excitation and Inhibition in Human and Monkey Neocortex

**DOI:** 10.1038/srep23176

**Published:** 2016-03-16

**Authors:** Nima Dehghani, Adrien Peyrache, Bartosz Telenczuk, Michel Le Van Quyen, Eric Halgren, Sydney S. Cash, Nicholas G. Hatsopoulos, Alain Destexhe

**Affiliations:** 1 Wyss Institute for Biologically-Inspired Engineering, Harvard University, Boston, MA, USA; 2 New England Complex Systems Institute, Cambridge, MA, USA; 3 NYU Neuroscience Institute and Center for Neural Sciences, New York University, NYC, NY, USA; 4 Laboratory of Computational Neuroscience, Unité de Neurosciences, Information et Complexité, CNRS, Gif-Sur-Yvette, France; 5 Institut du Cerveau et de la Moelle Epinière, UMRS 1127, CNRS UMR 7225, Hôpital de la Pitié-Salpêtrière, Paris, France; 6 Multimodal Imaging Laboratory, Departments of Neurosciences and Radiology, University of California San Diego, La Jolla, CA, USA; 7 Department of Neurology, Massachusetts General Hospital and Harvard Medical School, Boston, MA, USA; 8 Department of Organismal Biology and Anatomy, Committee on Computational Neuroscience, University of Chicago, Chicago, IL, USA

## Abstract

Balance of excitation and inhibition is a fundamental feature of *in vivo* network activity and is important for its computations. However, its presence in the neocortex of higher mammals is not well established. We investigated the dynamics of excitation and inhibition using dense multielectrode recordings in humans and monkeys. We found that in all states of the wake-sleep cycle, excitatory and inhibitory ensembles are well balanced, and co-fluctuate with slight instantaneous deviations from perfect balance, mostly in slow-wave sleep. Remarkably, these correlated fluctuations are seen for many different temporal scales. The similarity of these computational features with a network model of self-generated balanced states suggests that such balanced activity is essentially generated by recurrent activity in the local network and is not due to external inputs. Finally, we find that this balance breaks down during seizures, where the temporal correlation of excitatory and inhibitory populations is disrupted. These results show that balanced activity is a feature of normal brain activity, and break down of the balance could be an important factor to define pathological states.

It is believed that neuronal networks *in vivo* function in a “balanced” regime, where excitatory and inhibitory neuron activities maintain tightly correlated levels of activity. This balanced excitation/inhibition (E/I) was first suggested theoretically^1^,^2^ and later found experimentally *in vitro*^3^ and *in vivo*^4^. It is not only considered to be a functional cornerstone in the cerebral cortex, but also has been hypothesized to play a major role in areas other than cortex^5^.

Whether or not this concept of E/I balance can be extended to higher mammals, such as monkey or humans, is presently unknown. In this paper, we address this question by taking advantage of recent advances in the neural ensemble recordings with multi-electrode systems^6^ and the ability to separate excitatory and inhibitory cells^7^,^8^ in order to characterize the dynamics of excitatory and inhibitory populations, in human recordings (temporal cortex), and monkey recordings (motor and pre-motor cortex)^9^. The units were initially clustered based on spike shape, and in a next step, their excitatory or inhibitory character could be confirmed by their functional interactions, as determined using cross-correlograms^8^. To the best of our knowledge, this procedure provided the first coherent separation between identified populations of excitatory and inhibitory cells in humans. This was only possible because of the long period of the recordings (several segments of continuous 12-hour recordings for each subject). A similar discrimination between RS and FS cells was also done for the monkey recordings using a similar electrode array (see^9^). Together, these human and monkey recordings provide a unique data set where one can investigate the dynamics of excitation and inhibition in different brain states. In the present paper, we characterize the dynamics of ensemble inhibition and excitation at many temporal scales, analyze their interaction in different brain states and characterize the situations when the balance breaks down.

## Results

We first show the dynamic balance between excitatory and inhibitory cell activities in all different brain states, in human and monkey. We then use a number of methods to quantify this balance, as well as the deviations from balanced activity. Finally, we show an example of a pathological brain state where the balance breaks down.

### Recordings from different states are suggestive of excitatory and inhibitory balance

**Figure 1:**
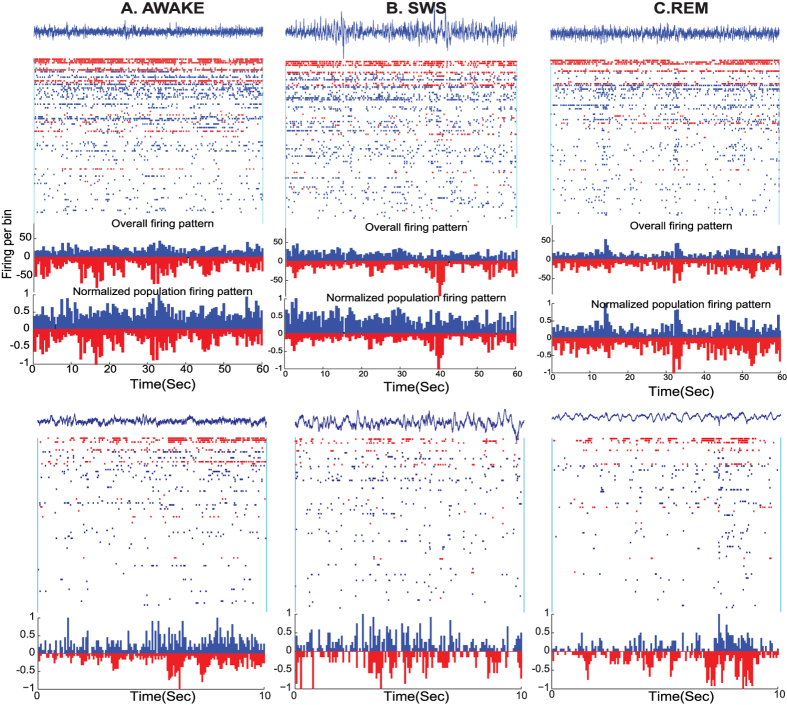
Sample recordings for awake (**A**), SWS (**B**) and REM (**C**) in human. Top row shows 60 second windows; bottom row shows a 10 second window of the same state. Putative inhibitory neurons (FS cells) are shown in red. Putative excitatory neurons (RS) are depicted in blue. At the top of each panel, a sample LFP trace (in blue) accompanies the spiking activity. Neurons are sorted based on their firing rate, within the portrayed epoch, in a descending order. Histograms show the overall activity of RS (blue) and FS (red) cells. In the normalized histogram, overall activity of each population is normalized to the maximum of firing rate (of the corresponding FS or RS population) in the shown example. Zero lag correlation values between the ensemble E and I are respectively: 0.726, 0.47 and 0.503.

Figure 1 shows local field potential (LFP) and unit recordings in human during different episodes of wakefulness, slow-wave sleep (SWS) and Rapid-Eye Movement (REM) sleep. The rasters of unit activity is divided into RS (blue) and FS (red) cells (see human and monkey RS/FS cells spike waveforms in Supplementary Fig. S1 and supplementary Fig. S2). We used this categorized ensemble activity to quantify the neocortical balance of excitation and inhibition.

A consistent observation for different states is that the ensemble inhibition and excitation mirror each other (Fig. 1 in humans; see also Supplementary Fig. S4 for monkey). One can see from the overall firing patterns (bottom), that in general an increase or decrease of the excitatory population is mirrored by similar dynamics among inhibitory cells, sometimes with a slight instantaneous deviation from balance (see below for quantification). Additionally, our analyses show that cells do not have a constant firing rate ratio throughout the recordings (supplementary Fig. S3). Thus the cells that show high firing rate at a given time period, do not necessarily have a higher firing rate. Variability of firing rate is a feature of both E and I subgroups. As the effect of high firing rate cancels out due to this variability, the estimates of the ensemble balance is not affected by a highly dominant group of cells.

**Figure 2:**
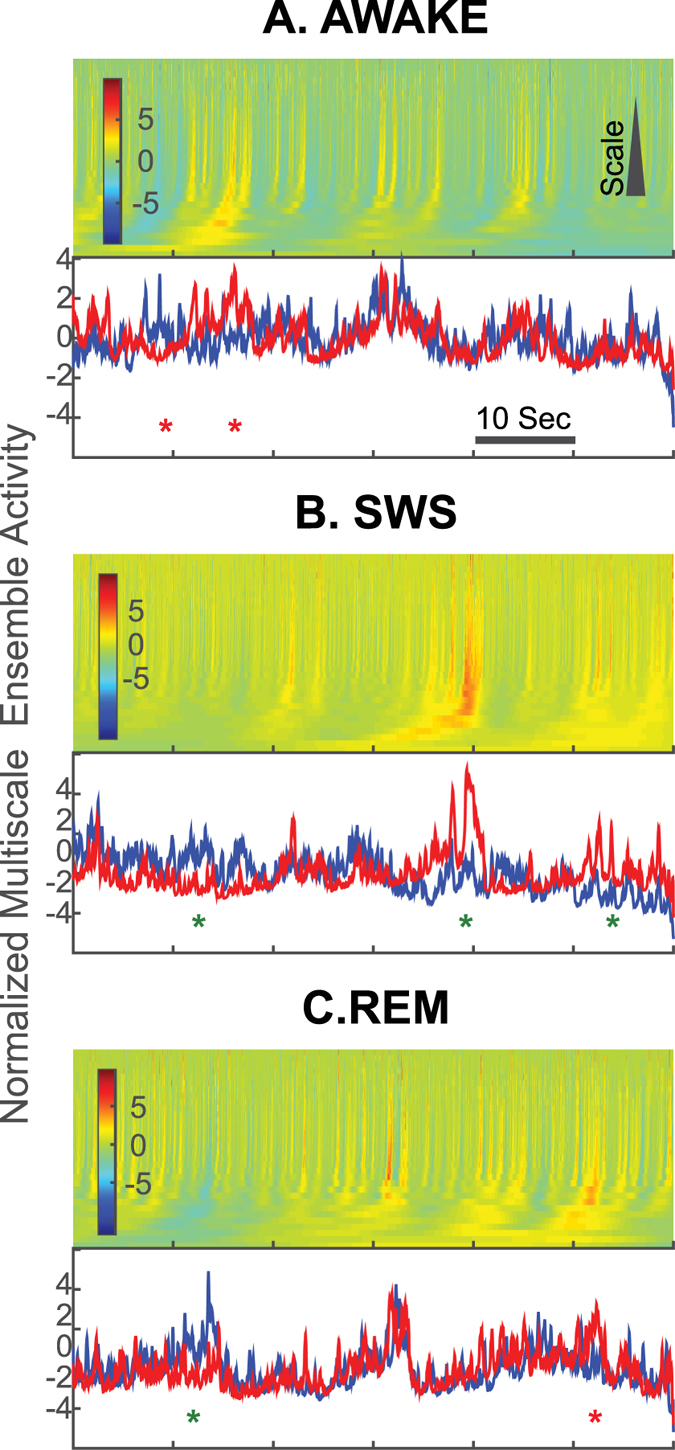
Balanced excitation and inhibition in sample 60 seconds recordings in human (The examples are from Fig. 1, panels (**A–C**)). Heatmaps at the top: Each row in the heatmap shows the normalized (Z-score) difference of ensemble excitation and inhibition for a given scale. The temporal scales are defined as time bins of different duration, increasing from the top to bottom (from fine-grain to coarse-grain). For details on coarse-graining, see methods. The color saturation towards red signifies instantaneous dominance of inhibition. Blue saturation shows instantaneous dominance of excitation, while green shows tight match between normalized ensemble excitation and inhibition. Bottom traces show the Z-scored addition of normalized excitatory (blue) and inhibitory (red) ensembles across the scales. Red stars show where the two interacting ensembles do not show similar trends in their multiscale fluctuations. Green stars show times when ensemble excitation and inhibition follow each other at certain scales but not all. Any of these states leads to a multiscale deviation from perfect balance. Such deviations are more pronounced for the SWS (see also Fig. 5).

A further look at the example recordings in Fig. 1 shows that most of the time, the two interacting ensembles follow the same trend at multiple scales and that deviations from perfect balance seem more pronounced for the SWS. Additionally, it is noticeable that sometimes the two ensembles follow each other at certain scales but not all (Fig. 2, bottom traces, representing the Z-scored addition of normalized excitatory (blue) and inhibitory (red) ensembles across the scales.). Similar patterns are observable in examples from monkey recordings (see supplementary Fig. S5). To test whether the analyses of those neurons that demonstrate very typical features of each (FS or RS) group, we chose the units only from the 30% of the two ends of the classification spectrum (as shown in supplementary Fig. S6A.). We then re-calculated the multiscale balance of E and I for the sub-set. At any given time t, we calculated the sum of the normalized difference of ensemble E and I across multiple scales. These values were turned into a histogram (as shown in supplementary Fig. S6B) to evaluate the distribution of dominance of E vs I. Instantaneous dominance of ensemble E is well balanced by instantaneous dominance of ensemble I throughout the whole recordings (supplementary Fig. S6B top row) as well as for the shown examples in supplementary Fig. S3 (supplementary Fig. S6B mid and bottom panels). This observation leads to question whether this E/I balance extends throughout all brain states, or if there are periods where the balance breaks down? In what follows, we aim to decipher such possible relations between excitation and inhibition at different temporal scales and provide a quantitative study of the dynamic aspects of E/I balance.

### Dynamic aspects of balance

An important property of the balanced activity is its multiscale aspect, as illustrated in Fig. 2. For each brain state represented, we calculated the difference between excitatory and inhibitory activities, represented as a function of the temporal scale considered. The multiscale aspect of the balanced activity clearly appears as the dominance of differences close to zero, with occasional deviations occurring transiently, especially in SWS.

We also evaluated the behavior of the correlations between excitation and inhibition across different temporal scales. As shown in supplementary Fig. S7, the ensemble FS and RS series showed well correlated dynamics. This type of ensemble correlation was observed across the multiple timescales. Further, the Monte Carlo randomization (four different types of randomization were implemented) showed that such correlation can not be due to aggregation of spike series into ensembles (For details of ensemble cross-correlogram and the randomization, see methods).

The observed ensemble temporal interdependence, was seen in different subjects with different number of FS and RS cells yet with the similar relative RS/FS count ratio of 4 to 1 (supplementary Fig. S7A1–A4), was multiscale (supplementary Fig. S7B1–B4), and was observed in all states (supplementary Fig. S7C1–C4). The percentage of co-occurrence of spikes (in the ensemble series) at the lag zero and the maximum observed percentage of co-occurrence (whether that maximum was at lag 0 or not) showed a robust multiscale linear relationship. A linear fit to the pooled values of lag zero vs maximum observed correlation, yielded a cross-subject average of 0.9988 ± 0.0134 for Awake, 0.9985 ± 0.0147 for REM, 0.9985 ± 0.0162 for light-sleep and 0.9977 ± 0.02539 for slow-wave sleep. We wish to emphasize two key findings: a) the maximum of the *ensemble* cross correlation is close to zero lag (note that this cross correlation is not calculated as an average of pair-wise cross-correlograms). Instead it represents the linear correlation of the two ensemble series (at different scales), on average, fire together and one cell population is not following the fluctuations of the group by some fixed delay. This does not necessitate the influence to be forced through a common input (see model of E/I balance below) another aspect is that as the data is coarse grained, the peak narrows and the higher correlation of the short delay shoulders (in comparison to long delays) dissipates. This phenomenon suggests that the instantaneous E-I relation (at the ensemble level) is tighter at coarse time scales.

### Model of E/I balance

**Figure 3:**
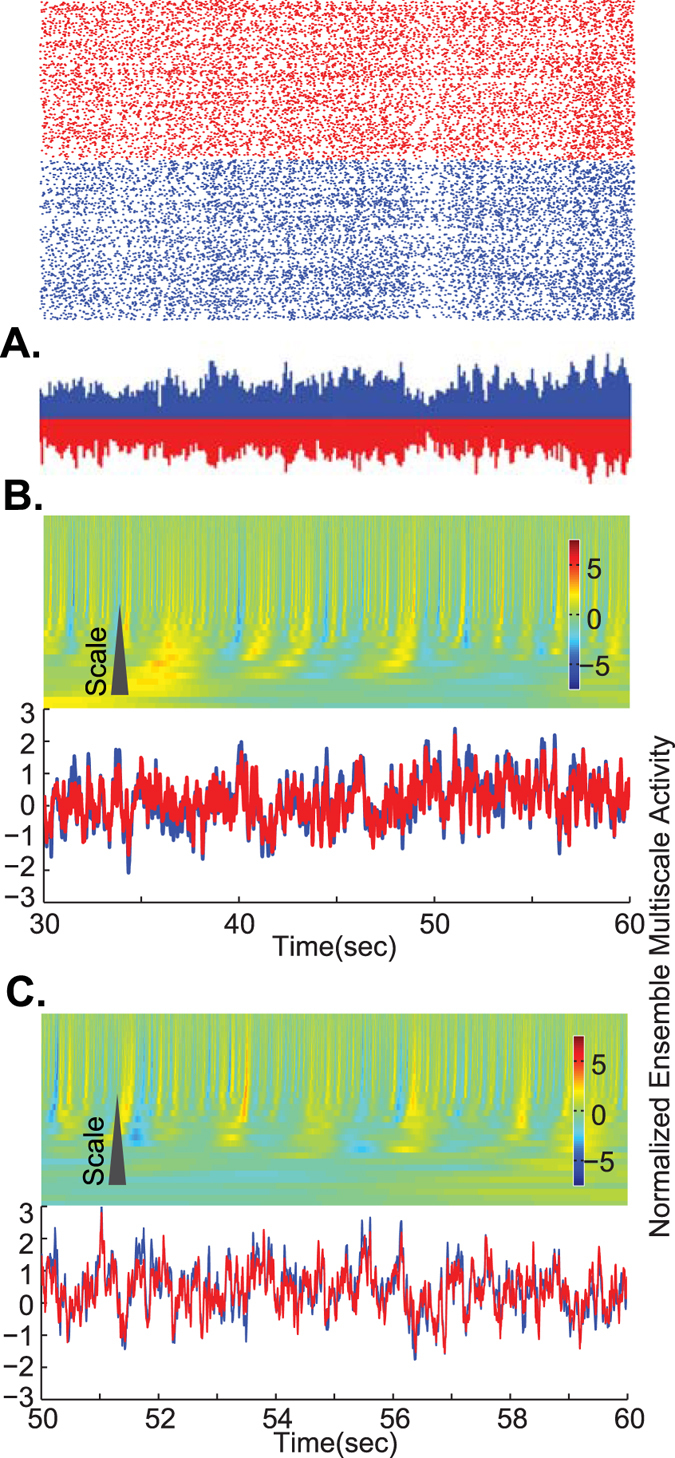
Multiscale balance in a computational model of AI (Asynchronous Irregular) states in networks of spiking neurons. (**A**) An example of raster plot and its normalized ensemble activity of COBA AI state. As in Fig. 2, preservation of excitatory-inhibitory balance across scales shows mirrored activity. (**B**) As in Fig. 2, the heatmap shows the normalized (Z-score) difference of ensemble excitation and inhibition for multiple scales. Line traces show the Z-scored addition of normalized excitatory (blue) and inhibitory (red) ensembles across the scales. (**C**) Same as (**B**) for a shorter period of time (last 10 seconds of (**B**)). These panels show that in general, the ensemble excitation and inhibition show an overall multiscale balance, even though there are instantaneous deviations from perfect balance.

To compare to a system with well-known and identifiable properties, we considered a network model of interconnected excitatory and inhibitory neurons displaying self-generated balanced activity states. This model^10^ consists of a conductance-based (COBA) network of 4000 neurons (2000 inhibitory and 2000 excitatory neurons; see Methods for details). In this model, the two population ensembles show a balanced mirrored activity (Fig. 3A). Further examination of the two populations shows that the overall balance is preserved across multiple scales (Fig. 3B,C and supplementary Fig. S8B). Similar to the experimental data, this is paralleled by the instantaneous deviations from perfect balance (Fig. 2, supplementary Fig. S5) and such observations are robust at many examined lengths of the data (see Fig. 2).

**Figure 4:**
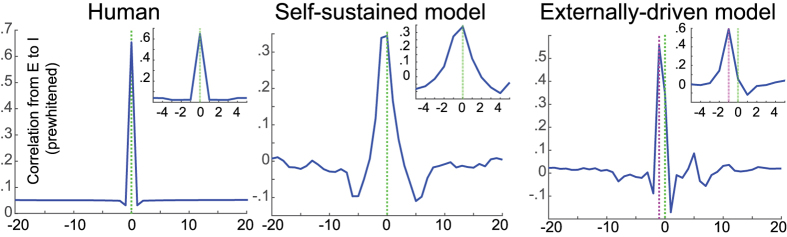
Correlation response analysis shows a comparative E:I impulse response for the finest time scale in Human data, self-sustained and externally-driven COBA models. In the externally-driven model, activity is mostly generated by the external inputs and stimulus is weaker on inhibitory cells. In contrast to Human data and self-sustained model, the E to I ensemble spiking correlation shows a shift from the central bin in externally-driven model. Insets show a zoomed in view.

To further probe the difference between model and data, we computed cross-correlations between excitatory and inhibitory populations using a similar procedure and data length as the experimental data (See Fig. 4, supplementary Fig. S8). Using prewhitened-based correlation analysis, we note that human and self-sustained COBA model show maximal correlation at central lag. However, when activity is mostly generated by the external inputs and stimulus is weaker on inhibitory cells, the correlation maxima shows a shift from the central bin (see Fig. 4 insets). Because these results may appear in contradiction with previous measurements of a lag between excitatory and inhibitory inputs in cortical neurons^5^,^11^,^12^, we investigated this issue in the model. We measured the *g*_*E*_ (excitatory conductance) and *g*_*I*_ (inhibitory conductance) inside 100 sample cells in the network during self-sustained activity to better portray the conductance correlation at the level of individual cell and ensemble spiking. In some cells *g*_*E*_ precedes *g*_*I*_, and in some *g*_*I*_ precedes *g*_*E*_. However, overall, *g*_*E*_ precedes *g*_*I*_ (~3 ms; see supplementary Fig. S9C). These simulations show that the precedence of excitatory over inhibitory inputs in single neurons is compatible with the fact that the excitatory and inhibitory neurons cross-correlation peaks at time zero, and thus there is no precedence of excitation over inhibition ensemble spiking. This absence of delay of inhibition suggests that the balanced activity observed in the data mostly stems from self-generated activity in the network, as in the model. In other words, this analysis suggests that the E/I balance is mostly generated by the local network through recurrent connections.

### Quantification of the E/I balance

**Figure 5:**
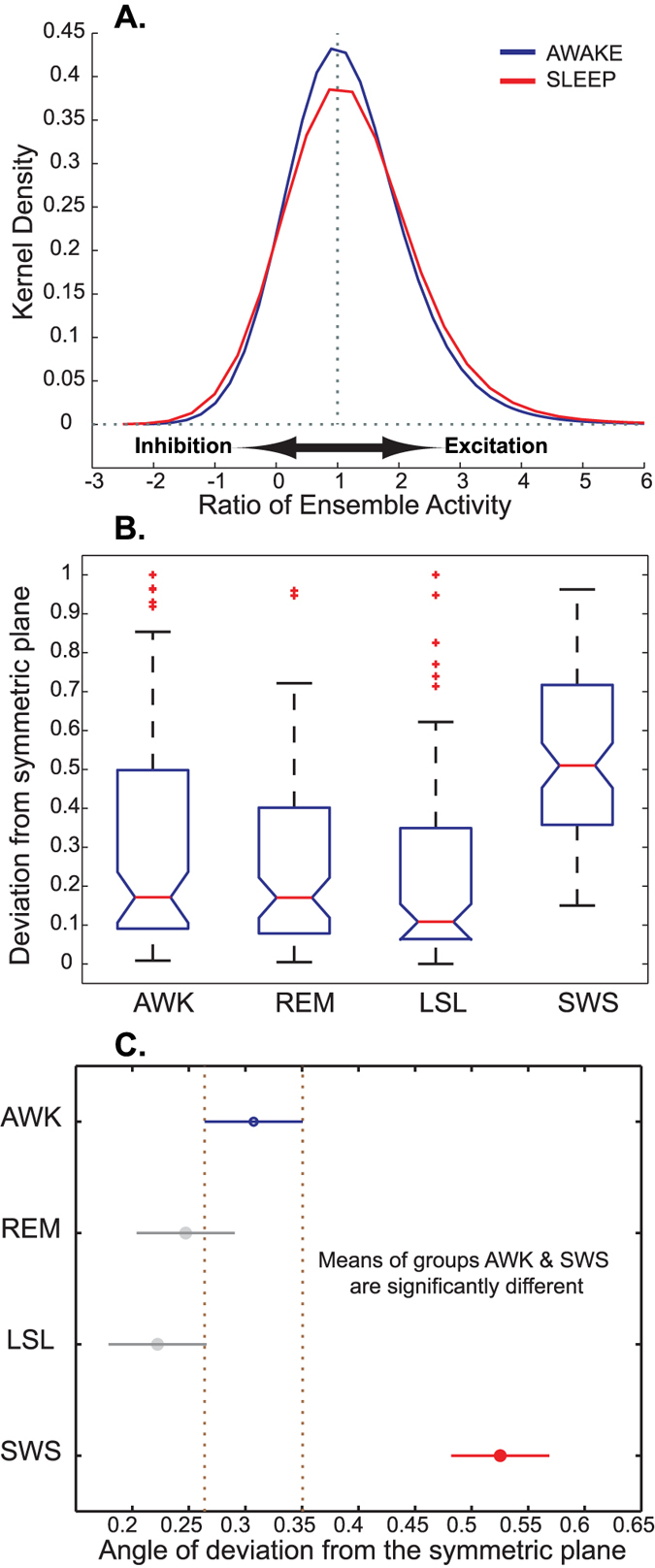
Panel (**A**), kernel density of the ratio of E/I for the monkey awake and sleep states. Perfect balance (where the magnitude of ensemble matches) would be represented by the vertical dotted line (=1). Though the qualitative symmetry in each state is preserved, the kernel density estimates of sleep and awake do not match, with more kurtosis in awake and broader shoulders in sleep. A Two-sample Kolmogorov-Smirnov test on the E/I ratio at the significance level of *α* = 0.01, rejected (*P*_*val*_ = 10^−4^) the null hypothesis that the data in awake and sleep are from the same continuous distribution. This is matched with the observations in humans, where the angle of deviation from the symmetric plane/axis is more pronounced during sleep rather than in awake (panel (**B**)). In the boxplot, the notch represents the median, the box boundaries show the lower and upper quartile SWS and the asterisks show the outliers. In a multiple comparison test, panel (**C**), awake and SWS show statistically significant differences between their means (*P*_*val*_ = 10^−3^). Note that only slow-wave sleep shows significant statistical difference with the other states at *α* = 0.05.

The quantification of the multiscale aspect of the balance (as in Fig. 2) shows that, although the E/I systems are generally balanced, there are occasional small deviations. We further quantified such deviations based on a quantification of the symmetry in joint E/I probability space (see Supplementary Fig. S10 and details in Methods). This analysis confirmed the multiscale investigation of Fig. 2A nonparametric two-sample Kolmogorov-Smirnov test, 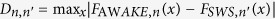 where 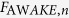 and 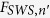 are the empirical CDFs (empirical cumulative distribution function) of the normalized E/I ratio distributions for the two states, rejected (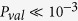) that they come from the same distribution at the significance level of *α* = 0.01. This shows that the degree of balance deviation is state-dependent. As also shown in Fig. 5, the highest degree of deviation from perfect balance happens during SWS.

This higher degree of deviation from balance during sleep could be attributed to the fluctuations of inhibitory/excitatory activity during up-state and down-state^3^,^13^,^14^,^15^, as hallmarks of a bistable regime where toggling between the two states is enforced by the mutual excitation and feedback inhibition^16^. Transient stability of both up and down states is the other side of the coin characterized by a rhythmic transition between quiescent and active states^17^. This property, leads to the observed higher degree of deviations from absolute balance plane.

### E/I imbalance during seizures

It has been speculated that the breakdown of the equilibrium between excitation and inhibition could lead to epilepsy. The idea that the lack of inhibition or excess of excitation can cause seizure is not a new one^18^. This has been experimentally used to induce or control seizures, such as for example by inducing inhibition using optogenetics^19^,^20^,^21^. Other optogenetic studies have related cortical E/I imbalance to other diseases such as mood disorders as well^22^. However, it has been argued that such a clear-cut idea of lack of inhibition or excess inhibition as the major frame of epileptogenesis is perhaps misleading^23^.

**Figure 6:**
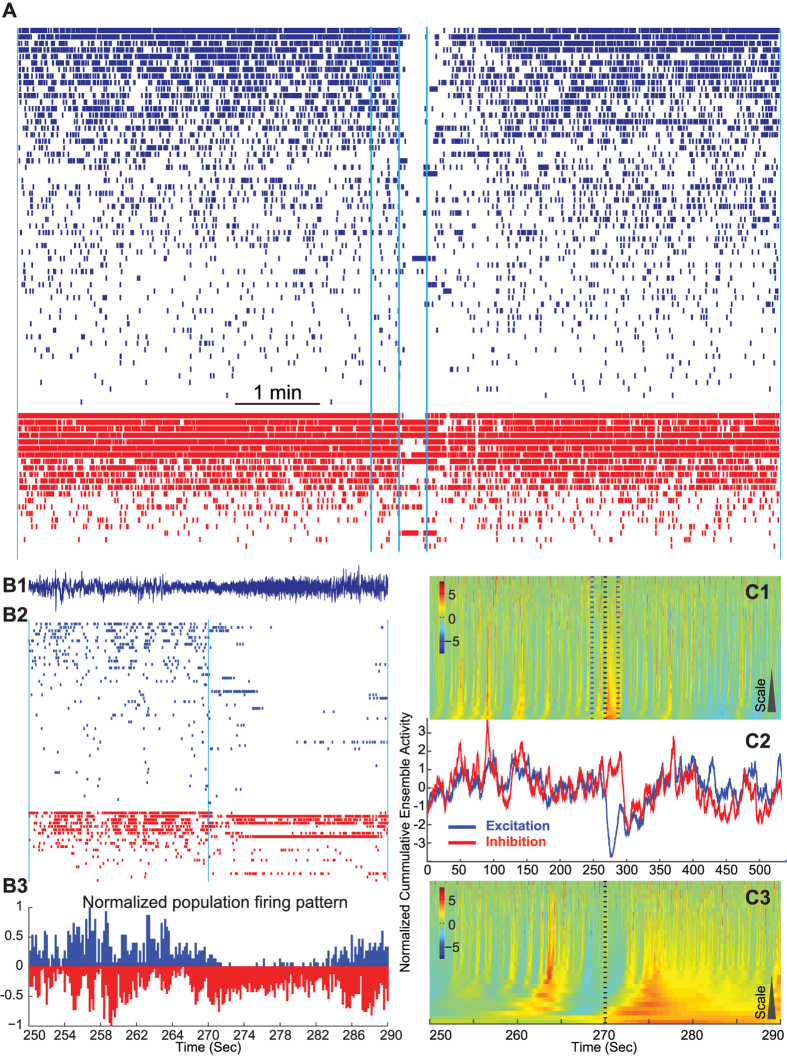
Misbalance in an example seizure recording in human. Panel (**A**) shows a 9 minute recording. Panels in B are the zoomed in version (the middle 40 seconds) of the same epoch (shown with the vertical lines in A). RS cells are in blue and ranked based on their firing rate within this epoch. Red cells show FS cells and are ordered according to their class firing rate. (**B1**) LFP activity in the zoomed period, corresponding to (**B2**) raster of FS and RS cells. (**B3**) Normalized mirrored histogram showing where the misbalance occurs. (**C1**) Heatmap of the normalized ensemble excitatory and inhibitory differences, corresponding to the 9 minute recording shown in A. Dotted lines mark the same boundaries as in (**B2**). (**C2**) Normalized cumulative ensemble activity of excitation vs. inhibition during the 540 seconds epoch. (**C3**) is the zoomed in version of the middle 40 seconds (corresponding to panels in B and the marked are by dotted lines in (**C1**). Seizure happens around the mid-point and is visually distinct from the rest of the recording. During the seizure, a clear misbalance occurs; however it shows complex multiscale characteristics (see supplementary Fig. S11 for more examples).

Here, we provide an example of a seizure recorded in one of our patients and show how E/I balance changes in a complex fashion that is in contrast to the simple misbalance scenario described above (see Fig. 6A,B). During the seizure some excitatory cells and some inhibitory cells increase their firing while some decrease or even stop firing^24^ yet an overall imbalance persists throughout the event. The same multiscale breakdown of the balanced excitatory-inhibitory activity was observed for all six seizures from two human patients. For additional examples, see supplementary Fig. S11.

To further elaborate on the quantification of misbalance during seizure, we tested the dynamics of the multiscale features of ensemble excitation and inhibition throughout the seizure. In the example seizure (shown in Fig. 6), there is a complete break-down of the balance, were the inhibitory cells initially dominate, which is further followed by re-emergence of balance toward the end of this seizure episode. Heatmaps of difference of normalized (Z-scored) ensemble excitation and inhibition, Fig. 6C1 and line plots of normalized ensemble excitation and inhibition Fig. 6C2, show that the interplay between the two populations harbors a multiscale feature during the misbalance.

Similarly, in other examples of seizures (supplementary Fig. S12), the two ensembles follow similar multiscale trends up to the seizure initiation (second 270), when suddenly the two systems become disengaged and fluctuate without any further interdependence. Such imbalanced fluctuation is later diminished and the two ensembles find their way to flow with the same multiscale trend again. Though this return to the balanced trend does not show any universal time scale. In some cases (Fig. 6C, supplementary Fig. S12D), it happens faster than others (supplementary Fig. S12A), and in other cases (supplementary Fig. S12B), it may not happen for even few minutes after the seizure has, electrographically, ended.

Note that the particular features illustrated here, such as the transient dominance of inhibition, may not be representative of all types of focal seizures (and a more detailed examination of the behavior of RS and FS cells during seizures is in progress (Ahmed *et al.*, in preparation). As these types of ensemble recordings become more abundant in clinical settings, in near future, it will become possible to test the multiscale features of balance in different types of seizures with the methods described here.

## Discussion

In this paper, we took advantage of the recent advances in the separation of excitatory and inhibitory cells, which were confirmed by direct cell-to-cell interaction^8^. Our present analysis demonstrates that the excitatory and inhibitory neural populations are balanced in the neocortex of human and monkey, as well as in a COBA (conductance-based) network model with AI (asynchronous irregular) properties. This overall balance extends to multiple temporal scales, as shown by the distributions of ensemble magnitudes (see Figs 2 and 3). We also found that the balance extends to nearly all brain states, and breaks down at multiple temporal scales during epileptic seizures (Fig. 6). The network recovers relatively quickly (tens of seconds) from the breakdown of balance after the end of the seizure (supplementary Fig. S12). This pattern suggests that the balance of excitatory and inhibitory activities is important for normal brain function and sleep, and its breakdown is associated to pathological activity, although no causal link could be established here.

Balance of excitation and inhibition has gone through many different renditions^11^, from simulations based on random walk models^25^, to opposing views of balanced synaptic input^1^,^26^ and later to those relating it to synchrony^27^, and those providing intracellular evidence for dynamic interplay between inhibition and excitation^28^,^29^. We showed here, for the first time, evidence for E/I balance in terms of network activity, estimated from large ensemble of units.

A particularly interesting finding is the absence of systematic phase lag between ensemble spiking of E:I populations, as shown by the zero-peaked averaged cross-correlation between RS and FS cells (see supplementary Fig. S7). This is intriguingly similar to network models where the balanced activity is self-generated (see Fig. 4), suggesting that the balanced activity is mostly generated by the local network, through recurrent connections. To better link this with the issue of external inputs, we have performed additional simulations of networks of excitatory and inhibitory neurons, and compared the case of an activity completely generated “internally” (self-sustained asynchronous-irregular states with no external input), with the same network with 10-times reduced collateral synaptic weights, but receiving a noisy external input. When the activity was self-sustained, the cross-correlation between E and I populations (determined from spiking activity) peaks at zero (Fig. 4). When network is externally-driven, ensemble spiking correlation peaks at a delayed lag (see Fig. 4 insets). These constitute further evidence that most of the balanced activity is generated internally by the local network.

In addition, we also found that overall, *g*_*E*_ precedes *g*_*I*_ in the self-sustained model (See supplementary Fig. S9). Prior studies have reported a lag between *g*_*E*_ and *g*_*I*_ as well^5^. A recent study also shows that this relation is state-dependent and the sign of the delay in anesthesia and awake are reverse^30^. This has been ascribed to the possibility that awake activity is more driven by the thalamocortical inputs whereas in anesthesia it is driven by internal states^31^. If so, the awake delay of *g*_*I*_ with respect to *g*_*E*_ could correspond to the feed-forward inhibition^12^. Like these studies, the asynchronous-irregular model presented here shows a delay between *g*_*E*_ and *g*_*I*_ (supplementary Fig. S9). The absence of delay in population spiking (in both experiments and model) along with the conductance delay at the level of individual cells suggests that most of the spiking activity is self-organized by the network. Future work should investigate in detail the computational properties of such locally-balanced networks, and how their dynamics can be shifted towards unbalanced pathological activities.

## Conclusion

Our results suggest that excitatory and inhibitory populations are tightly balanced across all states of the wake-sleep cycle, in both human and monkey. The only times where balanced activity breaks down is during epileptic seizures, suggesting that balanced activity is a fundamental feature of the normal functioning brain.

## Methods

### Recordings

Recordings of the ensemble neural activity were obtained through the implants of multielectrode arrays (Neuroport/Utah electrodes, Blackrock Microsystems). These arrays are composed of 100 electrodes arranged in a 10 × 10 matrix with an inter-electrode distance of 400 microns (for more details on electrodes see^32^,^33^). The patients, who were implanted, suffered from intractable seizures and were under neurosurgical monitoring to localize the focus of their epileptic seizure. The electrodes tips reached layer II/III of the neocortex (for details of implants see^24^). In the monkey, the implant was in the dorsal premotor cortex (PMd). Recordings were made during the performance of a motor task as well as during sleep (for details of implantation see^34^,^35^). For the human studies, patients were given consent forms with detailed description of the purpose of the study and its potential risks. Approval for all human experiments involving recordings of single unit activity in patients was granted by the Institutional Review Boards of Massachusetts General Hospital/Brigham & Women’s Hospital in accordance with the Declaration of Helsinki and required informed consent from each participant. For the primate experiments, all of the surgical and behavioral procedures were approved by the University of Chicago’s IACUC and conform to the principles outlined in the Guide for the Care and Use of Laboratory Animals (NIH publication no. 86–23, revised 1985; IACUC Approval number: 71565).

As has been described previously, the spikes of putative excitatory (Regular-Spiking., RS) neurons tend to be broader than putative inhibitory (Fast-Spiking., FS) neurons^7^,^36^. The recordings, were then spike-sorted and the units were categorized as either RS or FS. This categorization was based on morpho-functional characteristics of the spike-waveform and putative mono-synaptic connections (for details of such techniques see^7^,^8^). A variety of extracted features describing the shape of the average spike waveform were used, such as half-width of the positive peak, half-width of the negative peak, interval between negative and positive peaks (valley-to-peak) and the ratio of the negative to positive peak amplitude. Among the different criterion used to distinguish between cortical cell types in extracellular recordings, waveform duration is among the most reliable^7^. Some excitatory cells of motor may exhibit narrow spikes, but these are rare cases found in motor cortex^37^. In addition, some subtypes of inhibitory, non-fast-spiking interneurons show broad waveforms^38^. However, as these cells represent at most half of the GABAergic neuronal population^41^ and that, overall, inhibitory interneurons represent about 20% of all cortical neurons, the false positive rate of excitatory cell classification is at most 10%. Narrow-spike neurons encompass with high confidence various types of fast-spiking (and paravalbumin-expressing) neurons such as basket cells^39^. Based on these parameters, we classified the spike waveforms of all neurons into two groups using a standard K-means clustering algorithm. The procedure was repeated for each recording session separately; the neurons that were not assigned consistently to the same group were removed from further analysis.

### Multiscale temporal rescaling

We used 32 different time scales to remap the ensemble activity to renormalized time-series of excitation and inhibition. The scales were equally spaced in a logarithmic fashion between 1ms to 10938 ms. The logarithmic spacing was chosen for computational efficiency with respect to the number of scales, leading to a denser spread in finer time resolution. For this process, spikes of ensemble excitatory group were binned at different time-scales. As the number of excitatory and inhibitory neurons in each recording differs from one another (even though their relative size 4/1, is close to the anatomical observations), these values were normalized by the number of the neurons in each category of cells to obtain the ensemble fraction. This condition would overcome the limitations arising from both sub-sampling (here, 100 s of neurons out of many thousands) and spatial non-uniformity of sampling (although the recording electrode is a regular grid, unit recordings are not always regularly spaced). The same process was repeated for inhibitory neurons. The results yielded ensemble fractions of inhibition and excitation at many different time scales.

### Ensemble cross correlation

We first created the ensemble pool of the FS and RS cells in each subject of the study. The two series were lined up temporally along a common time axis. The ensemble RS and FS cells were used as the reference and target series respectively. For a given temporal scale, the bin length was defined according to the size of that scale as in Fig. 2. For each spike in the reference ensemble series, the delays of the spikes in the target ensemble series within −50 to + 50 bin lags were calculated. Next, the collective count of target spikes within a given lag was defined as the value of ensemble cross-correlogram between FS and RS series. This value was turned into a percentage for enabling the comparison across subjects with different number of neurons, multiple scales with a different number of aggregate of spikes, and different states with different duration of events. This process was realized for all scales.

### Randomization

Randomization was used to construct control for the ensemble cross-correlogram. We used four different systems of randomization to test for different within and between aspect of ensemble series. Any of the randomization protocols was realized 100 times. For each randomization category, the average of 100 random ensemble cross-correlogram was used as the control for verification of the observed patterns in the non-randomized cross-correlogram.

- *Random permutation of ISI in the ensemble series.* After pooling all the FS and RS cells into their ensemble series, the ensemble ISI (inter-spike interval) was calculated. Then, for each of the two ensemble series, a random permutation of its ISI was followed by cumulative summation of ISI, resulting in the new temporal order of ensemble spikes. This procedure guarantees that the randomized ensemble series has the exact number of spikes and exact set of ISIs as of the original ensemble series, albeit with different temporal arrangement of spikes within a given ensemble series.
- *Circular shift of spike ensemble.* In this type of randomization, the spikes were first pooled to create the ensemble FS and RS series. For each series, the ISI of the ensemble series was calculated. Then all the spikes in each series were shifted at once with a random value between the lower bound (1) and upper bound (maximum of the ISI in the ensemble series). In each randomization trial, it was made certain that the degree of the shift was not equal for the two FS and RS ensemble, guaranteeing that the temporal relation of the two series was never repeated. In contrast to the previous procedure (random permutation), this randomization kept the temporal of order of spikes within each ensemble series same as the non-randomized series. However, here the temporal relation of the two FS and RS ensembles was disrupted.
- *Fixed-ISI circular shift of spikes.* Before aggregating the spikes into the ensemble series, the ISI of each unit’s spike series was calculated. Then the spikes of the unit were shifted based on a random value drawn between the lower bound (1) and upper bound (maximum of the ISI of the that unit’s spike series). Next, all the randomized units were aggregated to create the randomized ensemble series. This procedure guarantees that the resultant ensemble series is constructed from units with intact internal structure of their spike timing but with a disrupted between-unit timing.
- *Local jitter randomization of spikes.* Next we tested the effect of randomization based on the statistics of each individual neuron before their aggregation to the ensemble series. First, the ISI of each FS (or RS) unit was calculated. Then the pool of the ISI as well as the ensemble of FS and RS was created. Next, each spike in the ensemble was shifted according a random number which was generated as the standard deviation plus a randomized (between −1 and 1, not including 0) multiple of the mean of pooled ISI. If the drawn random value was negative, the spike was shifted to the left and if the random chosen value was positive, the shift was toward the right in the ensemble series. This randomization, guarantees a tightly regulated data-driven local randomization based on the statistical properties of individual spikes.

### Deviation from absolute symmetry

We used complementary methods to calculate the deviation from symmetry between excitatory and inhibitory activities. First, we estimated the data-derived axis of symmetry based on the weighted bisquare robust regression. Then the angle between this axis and the identity line was used to represent the degree of deviation from pure symmetry. In parallel, for each time-scale, and for a given state, the time series of ensemble spiking data were reshaped into a 3-dimensional surface where the dimensions were the fraction of excitation, the fraction of inhibition, and number of their occurrences. As the durations of different states (SWS, REM, Wakefulness) differ from each other, the joint probability of ensemble fractions were also normalized by the whole length of the state to provide comparable results for further quantifications; i.e., the result is a surface in the 3D space of the fraction of excitation, the fraction of inhibition, and their joint probabilities. Theses surfaces were then Z-scored and their major orientation axis was calculated. Then the mid-point of the iso-surfaces along the major orientation axis was defined. Using orthogonal regression, a plane was fit to these point along the major orientation axis. This plane, is the plane of approximate symmetry of the data and divides the surface into two halves. In case of absolute balance at a given scale, the plane of symmetry of data would coincide with the symmetry plane of the 3D space. Deviations from perfect balance was calculated using the dihedral angle between the symmetry plane of data and symmetry plane of the 3D space. The results of the dihedral rotation was similar to the angle between axis of symmetry and the weighted least square regression (using robust bi-square fit) of the data in the 2D rendering of excitation fraction and inhibition fraction.

### Computational model

Network simulations were done using networks of excitatory and inhibitory spiking (integrate-and-fire type) neurons with sparse random connectivity (2000 excitatory and 2000 inhibitory neurons with 5% connection probability), and with conductance-based (COBA) synaptic interactions (See supplementary Fig. S8A). Such COBA networks were shown to display self-sustained asynchronous irregular (AI) balanced states (see (Vogels 2005) this reference for details of the parameters and see (Brette 2007) for codes). The network activity was entirely self-sustained (no added noise), after a kickoff random simulation to initiate the AI state. For creating an externally-driven network, we used the same method with 10-times reduced collateral synaptic weights, but receiving a noisy external input.

## Additional Information

**How to cite this article**: Dehghani, N. *et al.* Dynamic Balance of Excitation and Inhibition in Human and Monkey Neocortex. *Sci. Rep.* **6**, 23176; doi: 10.1038/srep23176 (2016).

## Supplementary Material

Supplementary Information
