# Supplementary material for: Dynamic Balance of Excitation and Inhibition in Human and Monkey Neocortex
Source: Sci Rep. 2016 Mar 16;6:23176. doi: 10.1038/srep23176 (PMC4793223; doi:10.1038/srep23176)
Supplement: Supplementary Information [file srep23176-s1.pdf]

Dynamic Balance of Excitation and Inhibition in Human and Monkey Neocortex

Nima Dehghani[1,2,*], **Adrien Peyrache**[3], **Bartosz Telenczuk**[4], **Michel Le Van Quyen**[5], **Eric Halgren**[6], **Syd S. Cash**[7], **Nicholas G. Hatsopoulos**[8], **and Alain Destexhe**[4]

[1]Wyss Institute for Biologically-Inspired Engineering, Harvard University, Boston, MA, USA

[2]New England Complex Systems Institute, Cambridge, MA, USA.

[3]NYU Neuroscience Institute and Center for Neural Sciences, New York University, NYC, NY, USA.

[4]Laboratory of Computational Neuroscience, Unité de Neurosciences, Information et Complexité, CNRS, Gif-Sur-Yvette, France.

[5]Centre de Recherche de l'Institut du Cerveau et de la Moelle épinière (CRICM), UPMC, Paris, France.

[6]Multimodal Imaging Laboratory, Departments of Neurosciences and Radiology, University of California San Diego, La Jolla, CA, USA.

[7]Department of Neurology, Massachusetts General Hospital and Harvard Medical School, Boston, MA, USA.

[8]Department of Organismal Biology and Anatomy, Committee on Computational Neuroscience, University of Chicago, Chicago, IL, USA.

[*]correspondence should be addressed to: nima.dehghani@wyss.harvard.edu, nima@necsi.edu

Supplementary Material

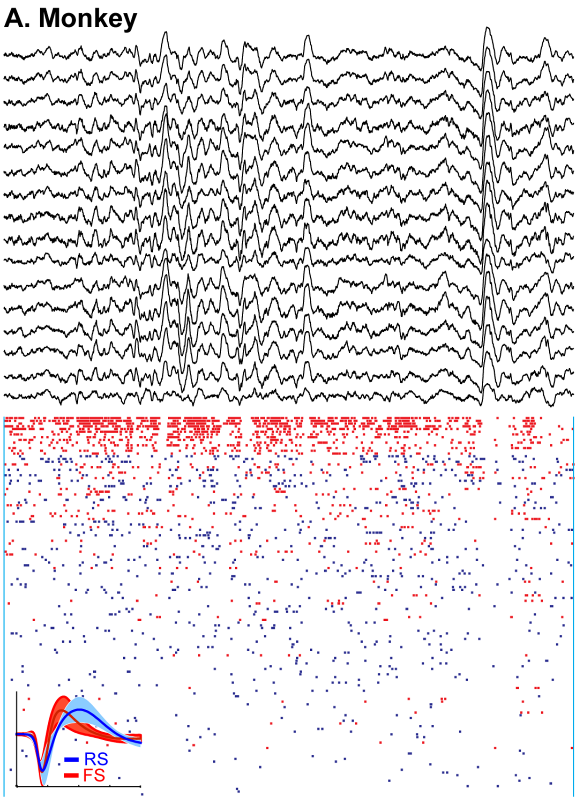

Figure S1. Sample recordings from a UTAH multielectrode array in Monkey (A) PMD and Human (B) Temporal cortices during 8 sec of SWS (Slow-wave sleep). In each panel, the upper section depicts LFP (local field potentials) from different locations of the multielectrode array. Lower sections show the corresponding Excitatory (blue) and Inhibitory (red) cells. Insets show the spike-waveform that was used to categorize the units into two inhibitory and excitatory cell populations.

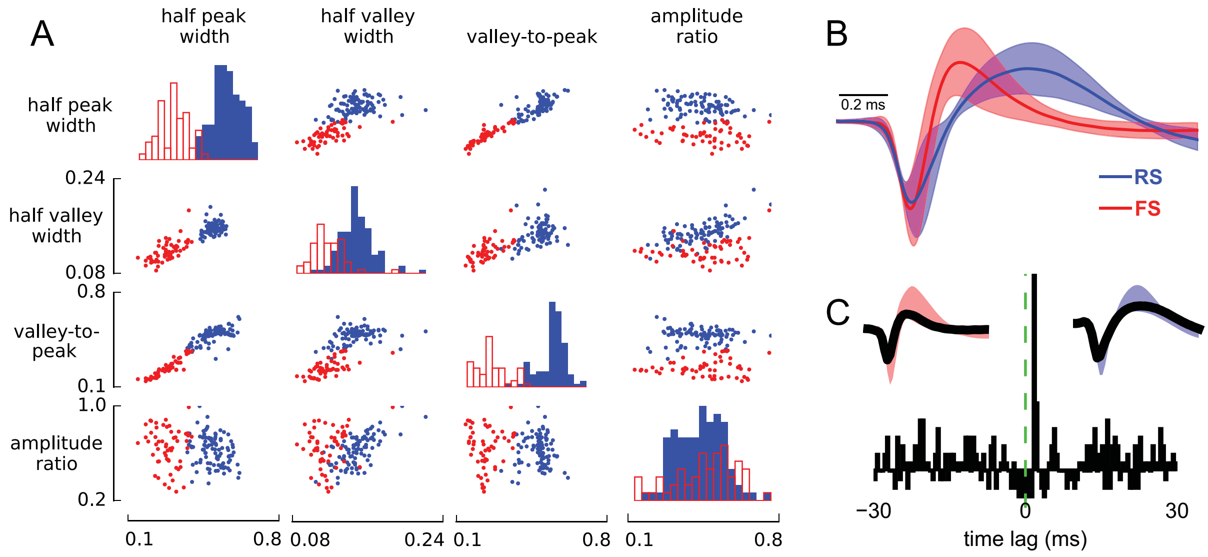

Figure S2. Separation between the putative regular spiking and fast spiking neurons in monkey. (A) Clustering based on 4 features of the spike waveform (half-peak width, half-valley width, valley-to-peak amplitude and valley-to-peak amplitude ratio) and we classified the spike waveforms in this 4-dimensional feature space into two classes (putative regular spiking, RS, and fast spiking, FS). The data points and histograms corresponding to these two types are shown in different colors (RS - blue, FS - red). The off-diagonal panels represent scatter plots of between each pair of features (labels at the top and left edges of the figure, each dot corresponds to a single neuron). The bar plots on the diagonal represent the histograms of spike-waveform features for each neuron type separately. (B) Average spike waveforms of the RS and FS type. (C) An example cross-correlogram (black bars, bin size 0.5 ms) between a pair of putative fast-spiking and regular-spiking neurons (spike waveforms shown in insets). The positive lags represent spikes of the FS neuron arriving after the spike of the RS neuron. The prominent peak at 2 ms corresponds to a presumed mono-synpatic connection from the RS neuron to the FS neuron, confirming functionally the separation based on electrophysiological features (spike waveform).

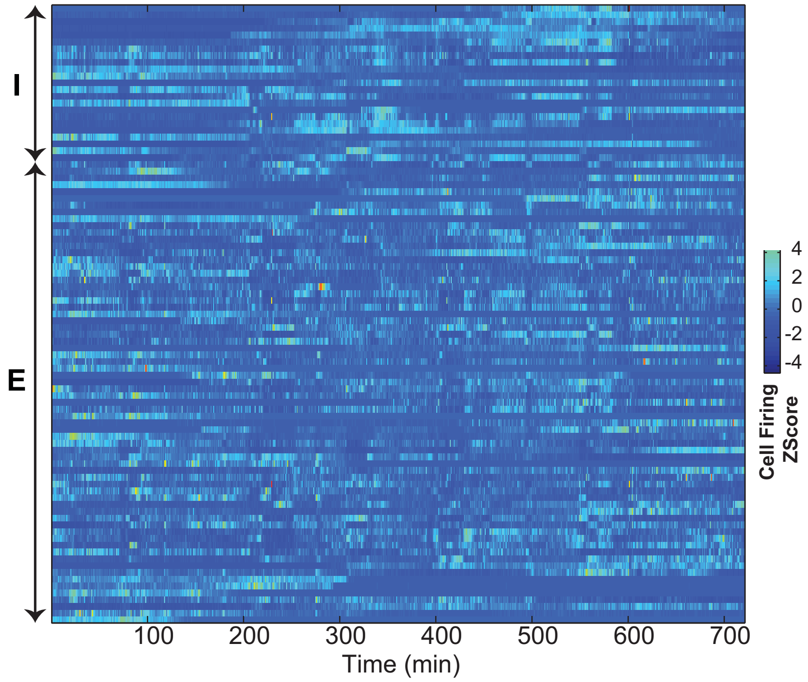

Figure S3. Firing variability during a 12 hour recording for I (N=23) and E (N=68) cells in human patient 1 (each cell firing cells is z-scored through the 12 hour recordings).

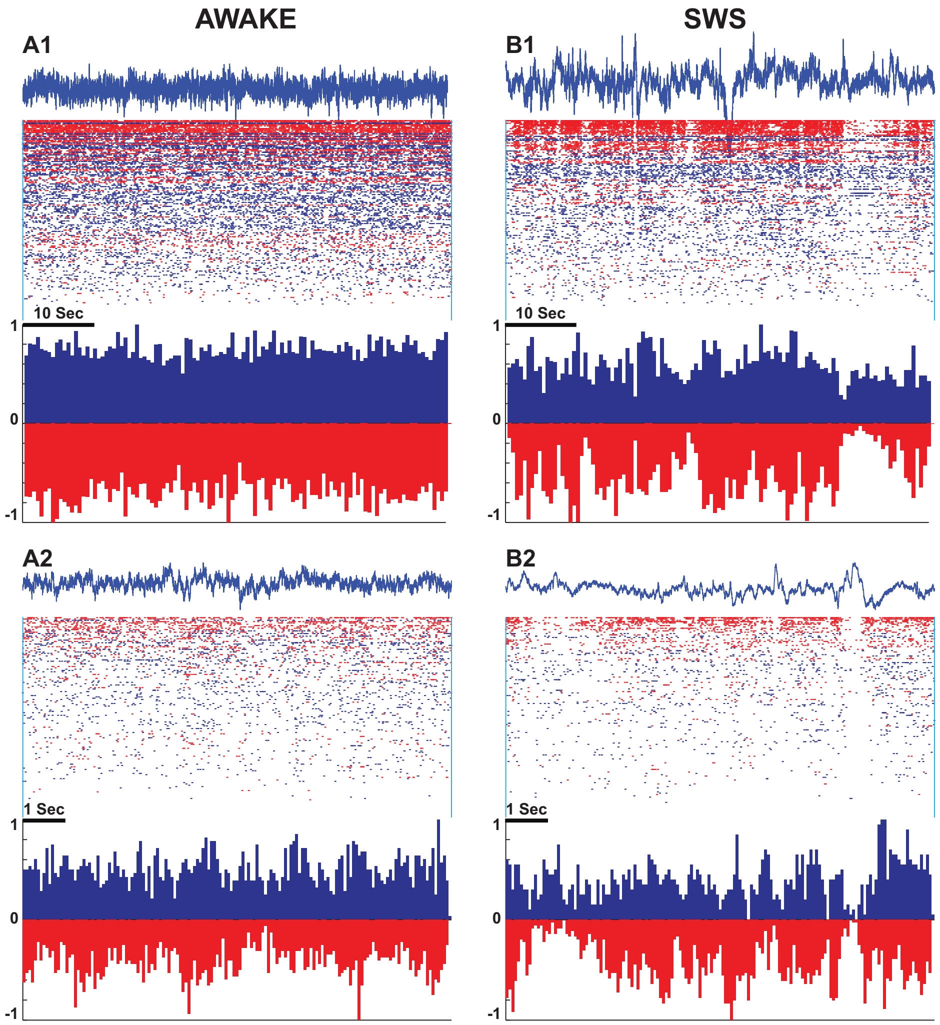

Figure S4. Sample recordings for AWAKE (left) and SWS (right) in monkey. A1 and B1 show 60 seconds windows; A2 and B2 show a 10 second window of the same state. In the rasters, putative inhibitory neurons (FS cells) and putative excitatory neurons (RS) are depicted in red and blue, respectively. In each panel, a sample LFP accompanies the spiking activity. Neurons are sorted based on their firing rate within the 60 sec epochs, in a descending order. Histograms show the overall excitatory activity normalized to the maximum of firing rate (within FS or RS category) in the shown example.

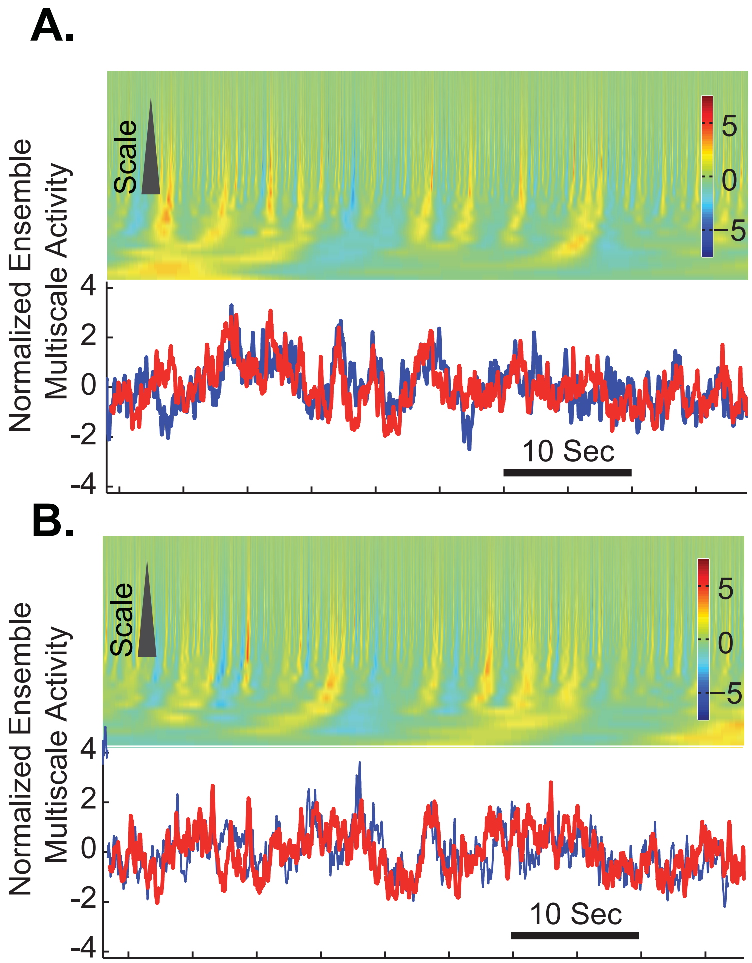

Figure S5. Multiscale features of excitation and inhibition balance in two sample recordings from the monkey. As in Fig.2, each row in the heatmap shows the normalized (Z-score) difference of ensemble excitation and inhibition for a given scale. The scales are defined as in Fig.2, increasing from the top to bottom (from fine-grain to coarse-grain). The color saturation towards red signifies instantaneous dominance of inhibition. Blue saturation shows instantaneous dominance of excitation, while green shows tight match between normalized ensemble excitation and inhibition. Line traces show the Z-scored addition of normalized excitatory (blue) and inhibitory (red) ensembles across the scales.

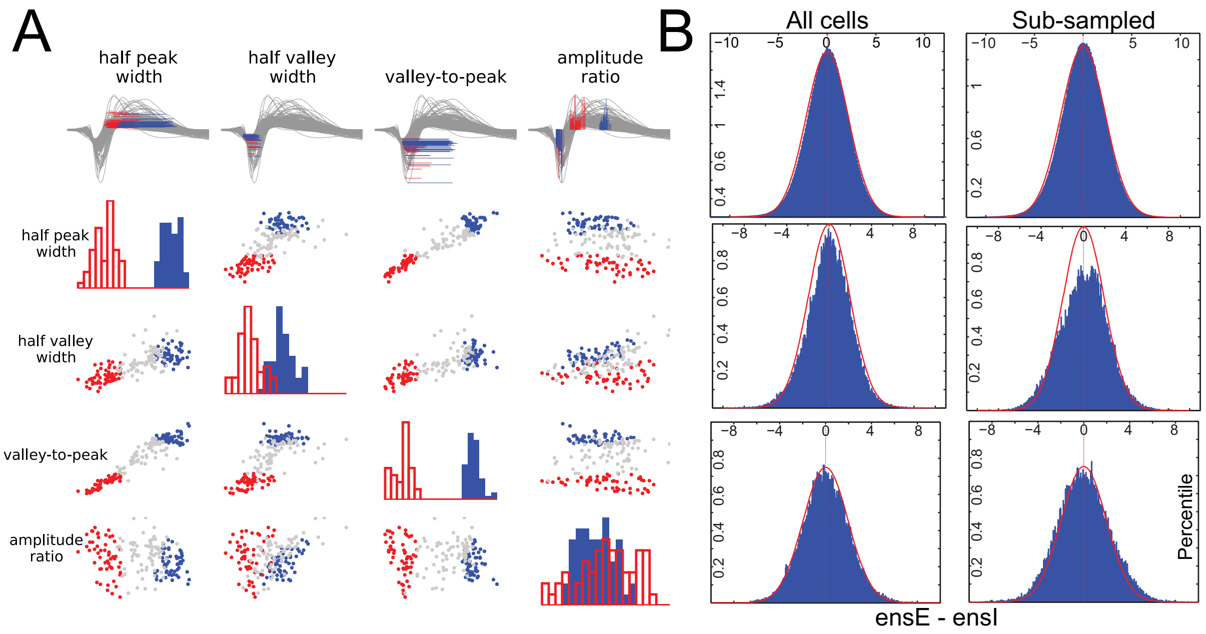

Figure S6. A. Scatter plots of pairs of spike-waveform features (off the diagonal) and histograms of individual features (on diagonal). The waveforms were clustered into RS (blue) and FS (red) types. Compared to Supplementary Fig.S2, we only take the waveforms with the 30% highest confidence of the clustering (the confidence being defined as the positive or negative projection on the vector connecting cluster center). B. Histogram of multiscale ensemble E and ensemble I difference at at given time t shows that balance of E and I is not affected by taking only the most clear FS and RS cells. The red curve is the scaled pdf of normal distribution calculated from the original data (left). Left column, original data. Right column, sub-set of neurons from 30% both ends of the FS-RS classification spectrum. Top row, whole data sets. Middle and bottom row, data in the segment from supplementary Fig.S5.A and B, respectively.

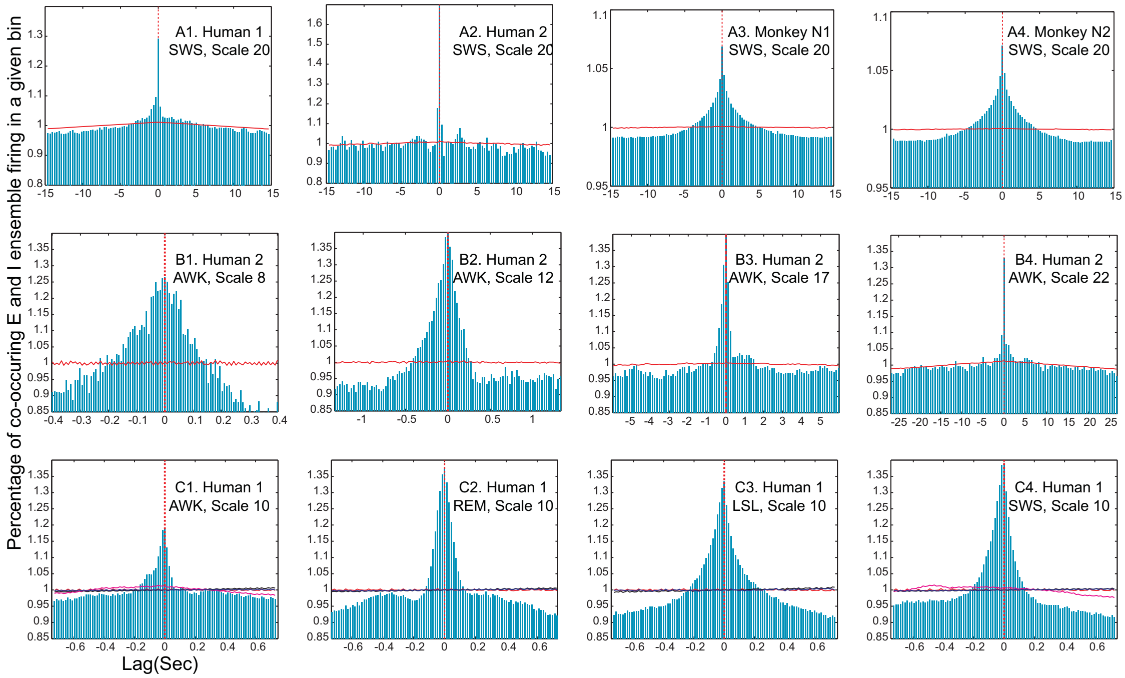

Figure S7. . Excitation and inhibition are correlated over multiple scales. In each panel, the cross-correlogram is shown as the histogram of delays of the spikes in the ensemble target series (inhibitory) with respect to the spikes of the reference series (excitatory). The vertical dashed line shows the lag zero, the horizontal line shows the average ensemble cross-correlogram of the Monte Carlo randomized process. In each histogram, the count of delays is turned into percentage (y-axis) for comparative reliability across different subjects (with different number of cells), different scales (different bin sizes) and different states (different length of the event). Note that in all panels lags -50 to +50 (bins) are shown. However, the span of time (x-axis, in sec) depends on the bin size of the evaluated timescale. A1 to A4, Ensemble cross-correlograms during slow-wave sleep across two different humans and two different nights of recording from the monkey are shown for a sample timescale. The shown randomized control (red) is the average of 100 realization of random permutation of the ensembles (see methods). B1 to B4, Ensemble cross-correlogram during wakefulness for a given subject across four different scales. Note that in each histogram of delays, the same number of lags (-50 to +50) are tested. The randomized control (red) is the average of 100 rounds of realization of random local jitter (see methods for details). C1 to C4, Ensemble cross-correlogram of different states in another human subject for an example scale. All four randomized controls (horizontal lines) show similar outcomes. The randomized controls show that these four different randomization procedures yield highly reliable dispersion of events in the ensemble series such that the ensemble cross-correlogram no longer shows any temporal interdependency between the ensemble excitatory and inhibitory series.

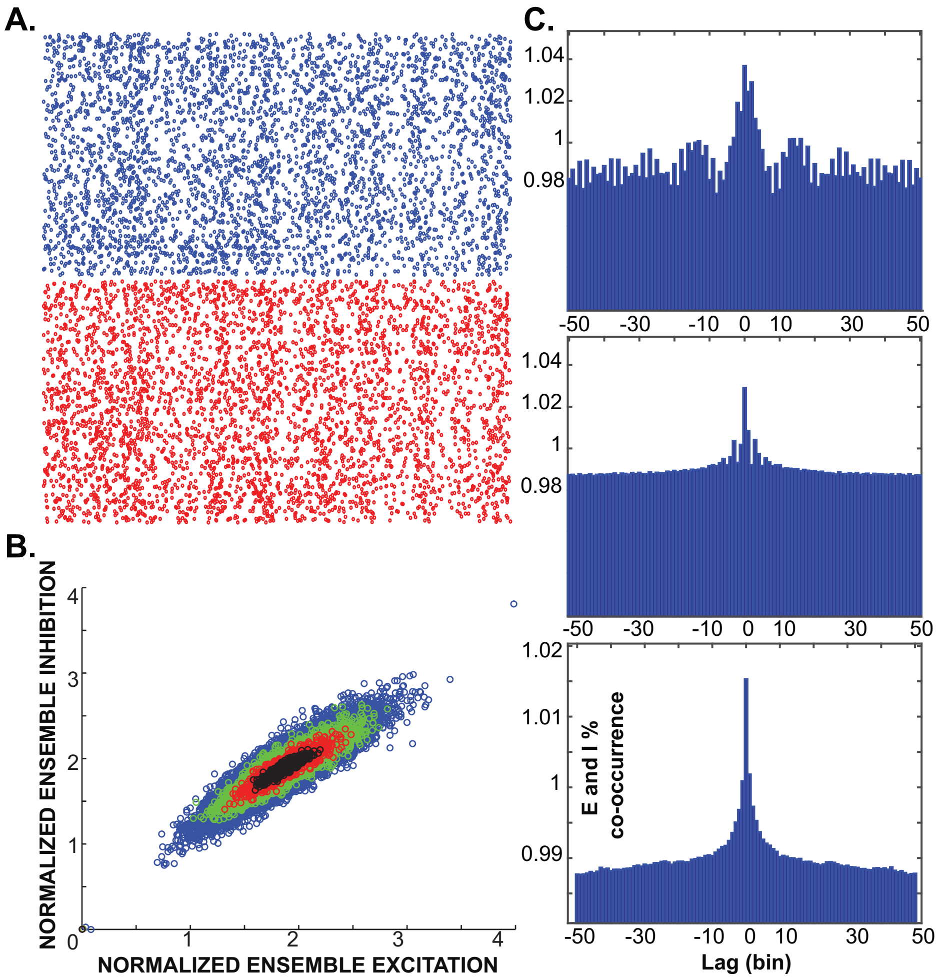

Figure S8. Balanced activity states in model networks of excitatory and inhibitory spiking neurons. A. Raster of 2000 excitatory (blue) and 2000 inhibitory (red) in a COBA (conductance-based) model, showing AI (asynchronous irregular) state for 100 sec. Panel B shows the scatter plot of normalized ensemble excitation and inhibition for times t_1 to t_n, with n representing the length of the time series at a given scale. Blue, red, green and black represent sample scales (comparable to scales used in the data; see Fig.2). Similar to the experimental data, the paired E-I data scatter along the diagonal line, representing an overall balance across multiple scales, with moment to moment dominance of E or I, while the degree of scattering along the diagonal shrinks with coarse-graining. C. Ensemble cross correlation score for three sample scales show preserved balance at central lag.

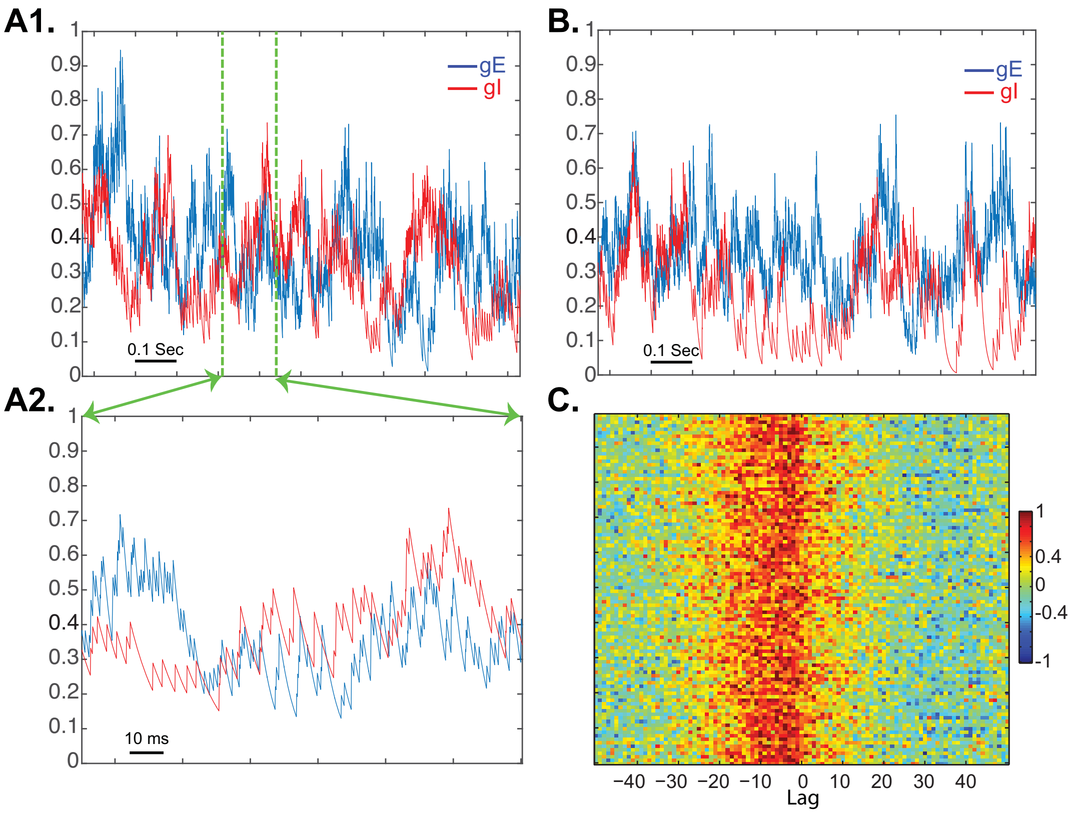

Figure S9. A1,B. Normalized g_E (excitatory conductance, blue) and g_I (inhibitory conductance, red) for a sample segments for two example E cells. A2, zoom in the marked region of A1. C. Correlation of g_E and g_I in 100 sample cells from the model. Each row shows normalized cross correlation between g_E and g_I for 50 lags (each lag = 1ms). Cells show variable conductance correlation maximum lag, with g_I lagging behind g_E on average. D. The cross-correlation between two exponential kernels provides similar characteristics to the g_E:g_I correlation. Inset, exponential kernels constructed with decay time based on average g_E and g_I rise time (across all cells) and delayed based on the average g_E:g_I conductance correlation.

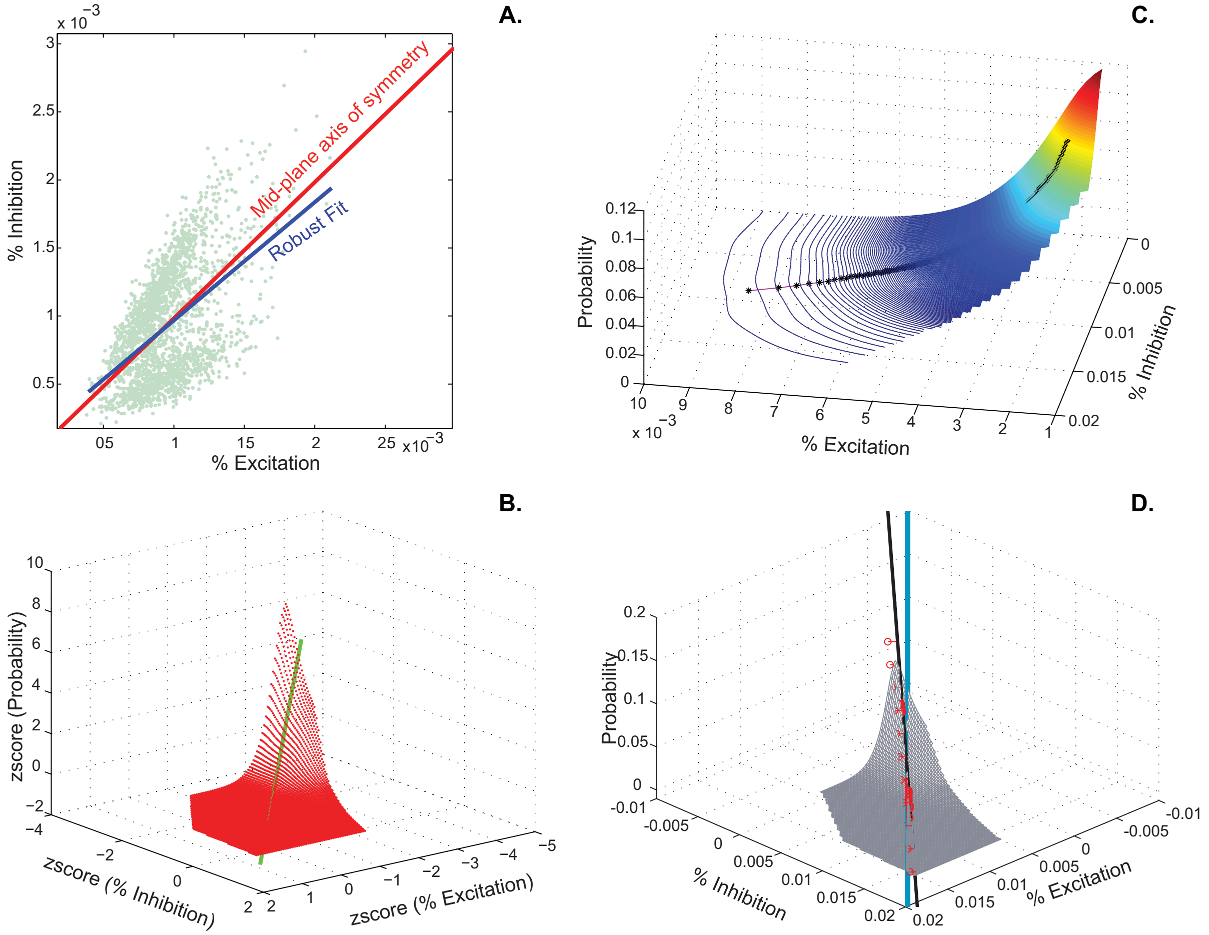

Figure S10. Panel A shows estimation of deviation from balance, between ensemble excitation and inhibition for a sample scale of SWS in a human subject, using robust bisquare regression. The fit (blue line) to the green cloud (data) shows the axis of symmetry of the data. Its deviation from the symmetry axis of the plane (in red) shows the degree of balance deviation. Panels B to D each show a different method for estimating the deviation from perfect symmetry. Panel B, shows the major orientation axis of the Z-scored data. Panel C, shows the distribution of E-I ensemble fraction pairs for a sample scale during SWS. The black lines are the centroids of the iso-surfaces. Panel D, combining these info, one can find the mid-plane of the data (shown in black) and find its tetrahedral angle with the plane of absolute symmetry (shown in cyan; see also the distribution of such angles in Fig.5).

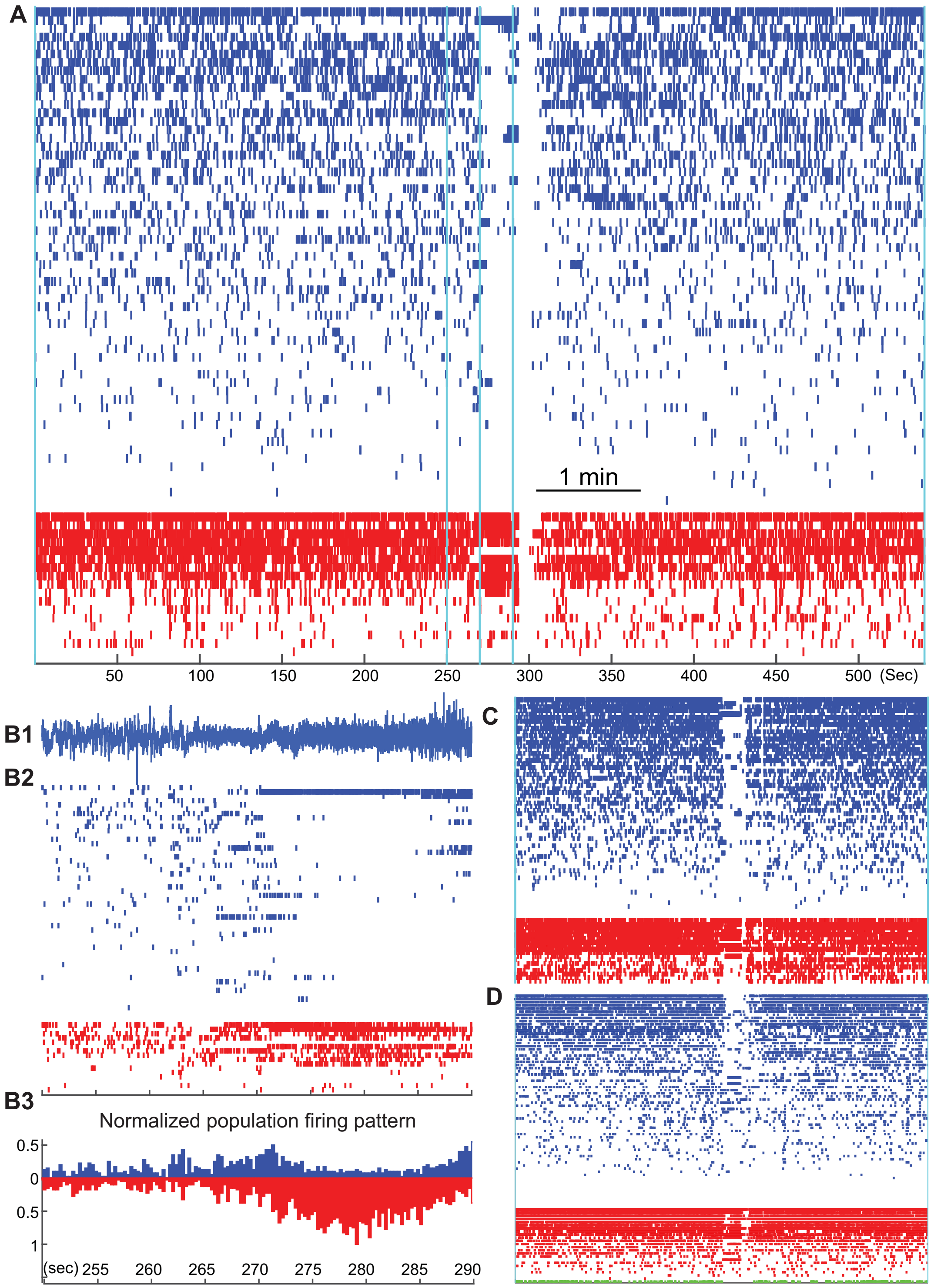

Figure S11. Misbalance in more example seizure recordings in human. Panel A shows a 9 minute recording. Panels in B are the zoomed in version (the middle 40 seconds) of the same epoch (shown with the vertical lines in A). RS cells are in blue and ranked based on their firing rate within this epoch. Red cells show FS cells and are ordered according to their class firing rate. B1, LFP activity in the zoomed period, corresponding to B2 raster of FS and RS cells. B3, Normalized mirrored histogram showing where the misbalance occurs. C, D. Two additional seizures similar to panel A (Note on panel D: in this patient, one unit was not categorized as either FS or RS, shown in green). Seizure happens around the mid-point and is visually distinct from the rest of the recording.

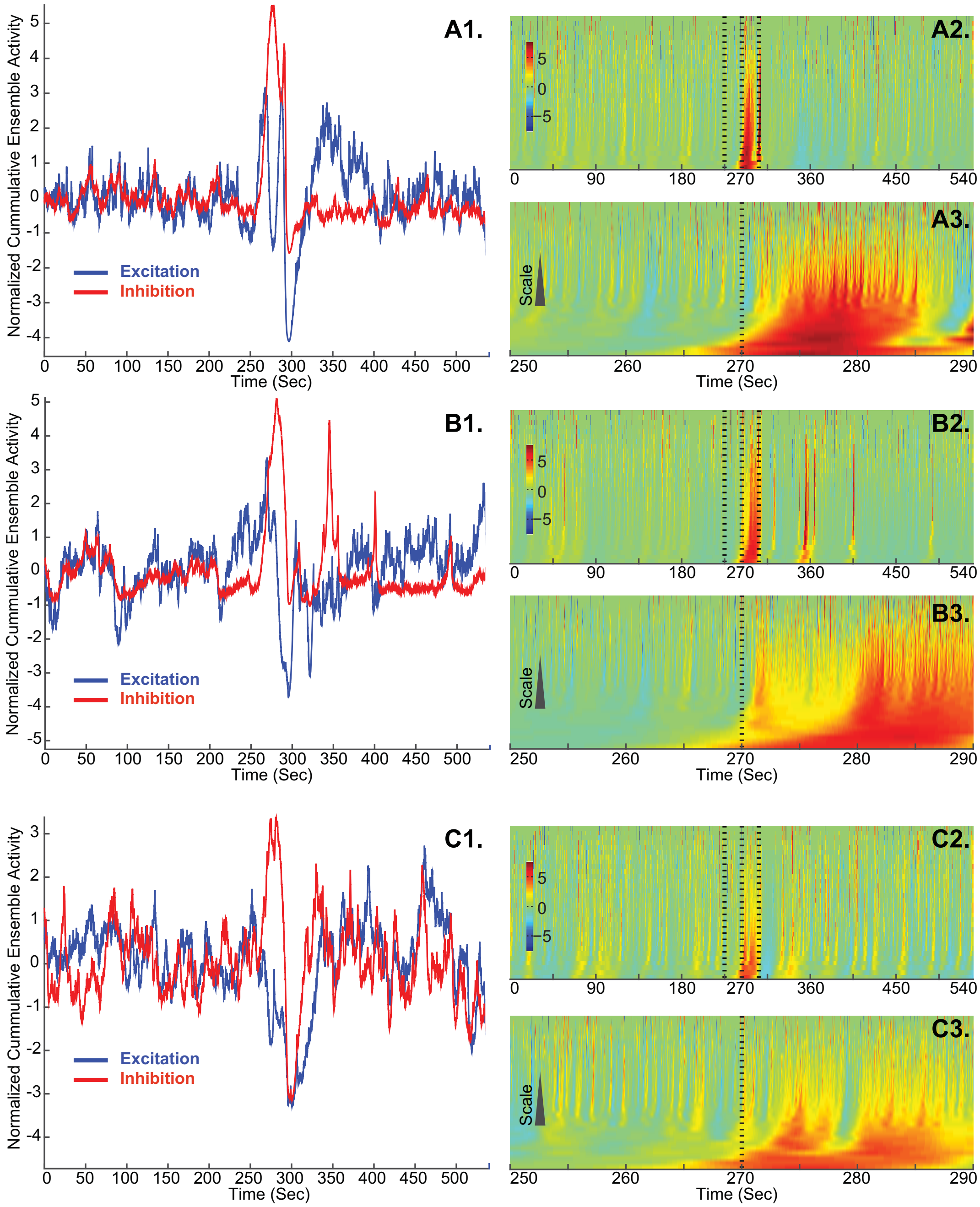

Figure S12. Break down of E/I balance in different seizure episodes. A1, B1 & C1. Multiscale features of balance breakdown during seizure. Blue and red traces show the normalized cumulative activity of ensemble excitation and inhibition across multiple scales (similar to Fig.2 bottom panels). In the shown examples (as well as in Fig.6), electrographic seizure starts around 270 sec. In all cases, ensemble excitation and inhibition follow the same multiscale trend. At the time of seizure, the two ensembles go through major fluctuations, and disentangle. In C1, return to multiscale balance trend happens fairly shortly. In A1, the system returns to balance a bit later (around second 400) and in B1, the system shows prolonged disturbed balance in the examined period shown here. Panels A2:3, B2:3 and C2:3 show the heatmap of the normalized ensemble excitatory and inhibitory differences, corresponding to the 9 minute recording and the middle 40 seconds zoom in (similar to Fig.6C).
